# A Study on Microstructure, Residual Stresses and Stress Corrosion Cracking of Repair Welding on 304 Stainless Steel: Part I-Effects of Heat Input

**DOI:** 10.3390/ma13102416

**Published:** 2020-05-25

**Authors:** Yun Luo, Wenbin Gu, Wei Peng, Qiang Jin, Qingliang Qin, Chunmei Yi

**Affiliations:** 1 College of New Energy, China University of Petroleum (East China), Qingdao 266580, China; guweibin1129@163.com (W.G.); pengwei1124@163.com (W.P.); jinqiangupc@163.com (Q.J.); 2 School of Petroleum Engineering, China University of Petroleum (East China), Qingdao 266580, China; 3 School of Automation and Electronics Engineering, Qingdao University of Science and Technology, Qingdao 266061, China; qqlqd@163.com; 4 Shandong Meiling Chemical Equipment Co., Ltd., Zibo 255430, China; sdmljsk@163.com

**Keywords:** 304 stainless steel, repair welding, heat input, residual stresses, stress corrosion cracking

## Abstract

In this paper, the effect of repair welding heat input on microstructure, residual stresses, and stress corrosion cracking (SCC) sensitivity were investigated by simulation and experiment. The results show that heat input influences the microstructure, residual stresses, and SCC behavior. With the increase of heat input, both the *δ*-ferrite in weld and the average grain width decrease slightly, while the austenite grain size in the heat affected zone (HAZ) is slightly increased. The predicted repair welding residual stresses by simulation have good agreement with that by X-ray diffraction (XRD). The transverse residual stresses in the weld and HAZ are gradually decreased as the increases of heat input. The higher heat input can enhance the tensile strength and elongation of repaired joint. When the heat input was increased by 33%, the SCC sensitivity index was decreased by more than 60%. The macroscopic cracks are easily generated in HAZ for the smaller heat input, leading to the smaller tensile strength and elongation. The larger heat input is recommended in the repair welding in 304 stainless steel.

## 1. Introduction

Repair welding is the most important means to repair the defects of aging pressure vessel and piping components, and is thus used for life extension [1]. Meanwhile, because of non-uniform heating and cooling during repair welding, the inhomogeneous plastic deformation and residual stresses are generated in the repair zone, which is the weakest area of the repaired structure [2]. The presence of residual stress has a harmful effect on strength [3], creep [4], fatigue [5], corrosion [6], and fracture [7], etc. The effects of repair residual stresses on structure integrity are greater than that of initial weld residual stresses [8]. Especially, during corrosive environment, stress corrosion cracking (SCC) phenomena easily occur in the repaired zone. Over 40% of weld repairs resulted in subsequent cracking, according to the industry survey conducted by EPRI [9]. Therefore, an improved understanding of the effect of repair welding on residual stresses and SCC behavior becomes critically important for performing structural integrity assessment of repaired equipment.

There have been numerous investigations employing experiment measurement techniques and numerical simulation methods for studying repaired residual stresses with various structural configurations. Dong et al. found that the tensile strength of repair was reduced by 30%–40% as compared to the initial welds due to the rather high level restraint in repair welds than initial welds [10]. The repaired residual stresses are varied for different material, component geometry, and welding processes, including the repair length, depth, the repair welding heat input, pass sequence, etc. Dong et al. [11] found that the transverse residual stresses transit from tensile stresses to compressive stresses near repair ends. Song and Shao [12] carried out an in-depth investigation of repair weld geometry effects on residual stress distributions and concluded that a weld repair should be designed as long as possible, as narrow as possible, and as shallow as possible. Jiang also found that the residual stresses are decreased with an increase of repair width [13]. The residual stresses are decreased with the clad metal thickness increases and base metal thickness decreases [14]. Using multiple-layer and high heat input weld could be useful for decreasing the residual stress [15]. With the increase of repair length, the transverse stress is decreased, and the longitudinal stress has little changes [16]. The repaired residual stresses can be either enhanced or weakened by change in repair length, depth, and width.

Except from residual stresses, the microstructure and microhardness of the repaired joint also played an important role in SCC incidents. Jiang et al. [17] investigated the effect of repair number on microstructure, hardness, and residual stress for a stainless steel clad plate. They found that the content of short ferrite and microhardness in weld increases as the repair number increases. The clad plate is recommended by repairing more than twice. Kessal et al. [18] concluded the hardness and residual stresses change with microstructure and ferrite content. Winiczenko et al. [19] studied the mechanical properties and microstructure of welded joint of ductile iron and stainless steel, the research results have good guidance to the friction welding of stainless steel. The corrosion resistance depends on the compressive residual stresses and the ferrite content, which are affected by the welding current and passes number. Reducing welding, machining, and surface strengthening technology can improve the corrosion resistance [20]. AghaAli [21] found that the HAZ of repair specimen is more sensitive to pitting corrosion and the corrosion sensitivity of HAZ was increased with repair number increases. As far as the corrosion resistance is concerned, repair welding can be safely employed as a means of extending the service life of the junction [22]. Raja et al. [23] compared the SCC behavior of as-welded and repair-welded joint while using the slow strain rate technique (SSRT). The SCC index for the repair welded joint is lower than that of the as-welded joint. Antunes et al. [24] also found that the corrosion resistance was depressed as the increases of repair number.

Although many careful investigations were performed on as-weld joint [25,26,27,28], there are few studies on the residual stresses and SCC behavior of repair welding joint by experiment. The repair welding usually increases the residual stress of the local repair welding area, and it has a certain influence on the mechanical property, SCC behavior, and service life of the repaired area. The current large number of literature is mainly concentrated on the study on the effects of repair welding length, width, and times on structure integrity and pitting corrosion behavior, and little attention was paid to the effects of repair welding heat input and reinforcement height. It is still unclear how the repair welding heat input and reinforcement height affects the microstructure, residual stresses, and SCC behavior of repaired joint. Therefore, the aim of this study is to study the effects of repair welding technology (heat input, reinforcement height) on microstructure, residual stresses, and SCC behavior of repair welded joint, which will be of great significance for prolonging the service life of the repair welded equipment.

This work is reported in two parts. The effects of heat input and reinforcement height were discussed in Part I and II, respectively. The heat input is an important welding parameter for ensuring the structure integrity of welded joints. An increase of welding heat input can reduce the occurrence of inadmissible welding imperfections in welded joints [29]. In general, with the increases of heat input, the molten zone and HAZ are broadly spread, and the maximum temperature welding pool is also increased. Too larger heat input might generate a harmful effect on the structure integrity of welded joint. For example, the residual stresses for dissimilar pipe weld joint are increased as the welding heat input increases [30,31]. The impact toughness was found to be decreasing [32] and the corrosive insensitivity increased [33] with an increase in heat input. Ravisankar [34] pointed out that the immediate and appropriate heat input was found to give better weld penetration with controlled molten zone and good mechanical properties. The effect of heat input on repair weld joint is different from that on first weld joint due to geometry constraint [10]. Thus, the effects of heat input should be investigated in detail for every types of welded joint. The finite element modeling (FEM) is a good method for clarifying the effects of heat input on the weld pool and maximum welding temperature before welding trail [35,36,37,38]. Jiang et al. [39,40,41,42,43,44,45,46,47,48] made a larger number of investigations of welding residual stress by FEM, and some key method of reducing residual stresses are obtained.

In this paper, Part I, the effects of repair welding heat input on microstructure, residual stresses, and SCC of 304 stainless steel were investigated by FEM and experiment. Part I starts with a brief description of the finite element residual stress modeling procedure that was adopted in this study. Subsequently, the experimental procedure was shown and finite element results were verified. Afterwards, a finite element analysis and experiment results are presented for identifying the effect of heat input. Finally, an optimized repair welding heat input technology was proposed.

## 2. Finite Element Modeling

### 2.1. Geometry Model

**Figure 1:**
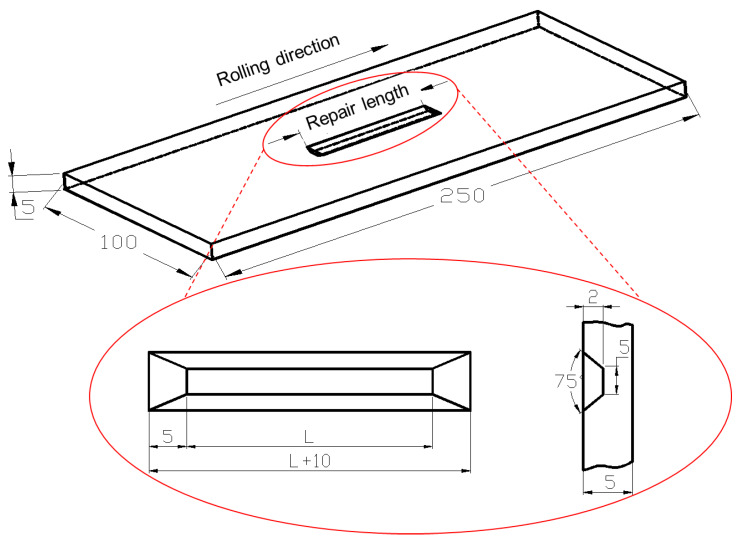
The schematic diagram of repaired specimen.

The 304 stainless steel plate was repair welded by A102 electrode. The dimension of parent specimen is 250 mm × 100 mm × 5 mm. The groove defect is extracted by machining in the center of the sample in order to make the surface defect. Figure 1 shows the schematic diagram of repaired specimen. The defect depth is 2 mm and the groove angle is 75°. The width defect bottom is 5 mm. L = 40 mm is the repair welding length.

**Figure 2:**
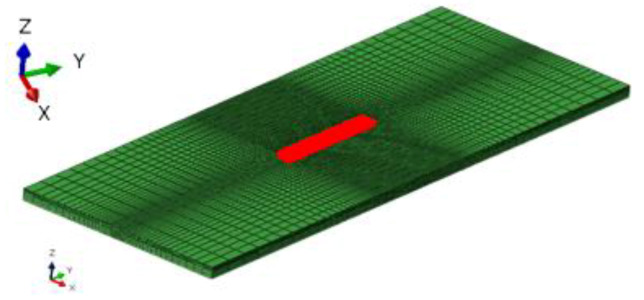
Finite element meshing of repaired specimen.

A three dimensional (3D) model was built according to the dimension that is shown in Figure 1. Figure 2 shows the typical finite element meshing of whole repair specimen. The meshing is dense in the weld, and then it becomes coarse gradually far away, in order to reduce the computation time. The element types for the thermal analysis and mechanical analysis are three-dimensional eight node linear heat transfer element (DC3D8) and three-dimensional eight node linear brick reduced integration element (C3D8R), respectively.

### 2.2. Simulation of Residual Stress

Here, the initial weld residual stresses are assumed to be negligible in repair weld modeling. Repair welding simulation is composed of welding temperature analysis and then followed by stress analysis, which employs the temperature that was obtained from thermal analysis. A sequential coupling analysis was used for the finite element analysis. The element birth and death technique were adopted to simulate the weld bead deposition by ABAQUS code (6.10).

#### 2.2.1. Thermal Analysis

In thermal analysis, the welding process is primarily simulated by applying a heat source for the double ellipsoidal distribution, which is expressed by the following Goldak equations [49]:

For the front heat source:

(1) $$q(x,y,z,t)=\frac{6\sqrt{3}f_{f}Q}{abc_{1}\pi \sqrt{\pi }}e^{-3x^{2}/a^{2}}e^{-3y^{2}/b^{2}}e^{-3{\left(z-vt-z_{0}\right)}^{2}/c_{1}{}^{2}}$$

For the rear heat source:

(2) $$q(x,y,z,t)=\frac{6\sqrt{3}f_{r}Q}{abc_{2}\pi \sqrt{\pi }}e^{-3x^{2}/a^{2}}e^{-3y^{2}/b^{2}}e^{-3{\left(z-vt-z_{0}\right)}^{2}/c_{2}{}^{2}}$$

where *f_f_* and *f_r_* give the fractions of the heat deposited in the front and the rear parts, respectively. It assumes that the *f_f_* is 1.5 and *f_r_* is 0.5 when considering that the temperature gradient in the front leading part is steeper than that of the tailing edge. *Q* is the power of the welding heat source and the *z*_0_ is the position of the heat source in z-direction when *t* is zero. *x*, *y*, and *z* are the local coordinates of the double ellipsoid model aligned with the welded plate. The heat source of double ellipsoidal distribution for the moving welding arc is modeled by a user subroutine DFLUX in ABAQUS compiled by FORTRAN program.

Convection (*q_a_*) and radiation (*q_r_*) in the welding and subsequent cooling are both taken into account, which is simulated by:

(3) $$q_{a}=-h_{a}(T_{s}-T_{a})$$

(4) $$q_{r}=-\varepsilon k[{\left(T_{s}+273\right)}^{4}-{\left(T_{a}+273\right)}^{4}]$$

where *h_a_* is convection coefficient (15 W/m^2^K) and *ε* is emissivity (0.8); *k* is the Stefan–Boltzmann constant (5.67 ×10^−8^ J/(m^2^K^4^s). *T_s_* and *T_a_* are the current and room temperature (20 °C), respectively. Table 1 lists the chemical compositions of the 304 stainless steel. Additionally, Table 2 lists the temperature-dependent thermal physical properties of 304 stainless steel.

#### 2.2.2. Residual Stress Analysis

The residual stress is calculated by using the temperature distribution that was obtained from thermal analysis as input data. For 304 stainless steel, there is little change on microstructure, thus solid-state phase transformation does not occur. Therefore, the total strain can be decomposed into three components, as follows:

(5) $$\varepsilon =\varepsilon^{e}+\varepsilon^{p}+\varepsilon^{ts}$$

where *ε^e^*, *ε^p^*, and *ε^ts^* stands for elastic strain, plastic strain, and thermal strain, respectively. Elastic strain is modeled while using the isotropic Hooke’s law with temperature-dependent Young’s modulus and Poisson’s ratio. The thermal strain is calculated using the temperature-dependent coefficient of thermal expansion. For the plastic strain, a rate-independent plastic model is employed with Von Mises yield surface, temperature-dependent mechanical properties, and isotropic hardening model, which is commonly used for weld simulations due to the easily-conducted set of tests required for model calibration. The annealing effect is also considered here, and the annealing temperature is assumed to be 800 °C for all of the materials in the present model. For stress analysis, temperature-dependent mechanical properties of the materials are incorporated, as shown in Table 3. During the stress analysis, four end nodes on bottom surface are constrained in order to avoid the rigid body motion.

## 3. Experimental Details

### 3.1. Sample Preparation

**Figure 3:**
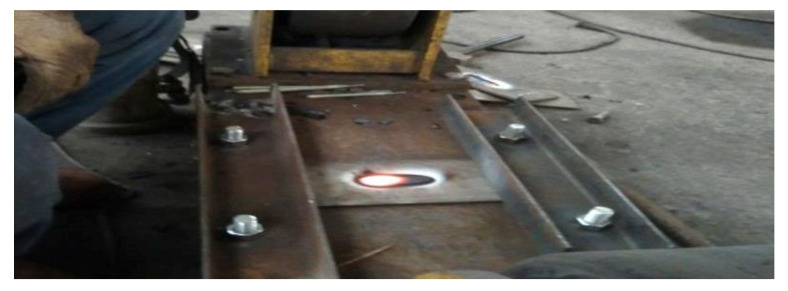
The constraint in the process of repair welding.

Manual arc welding technology is used to repair the defects of the sample. Before repair welding, the defects are cleaned to prevent impurities from entering the repair layer, and then preheating the areas around defects with 200 °C in order to keep the base metal dry during welding. In the process of repair welding, fixtures should be used to fix both ends of the sample in order to prevent excessive deformation of the specimen, as shown in Figure 3. After repair welding, the sample was naturally cooled in air.

Table 3 lists the welding parameters of repaired specimens with different heat inputs. The repair length is 40 mm. The heat input of specimen A1, A2, A3, and A4 are 4.20, 4.67, 5.13, and 5.60 kJ/cm, respectively. The heat input is calculated by equation, as follows:

(6) $$q=\frac{U\cdot I}{v}\cdot \eta$$

where *q* is heat input; *U* is welding voltage; *I* is welding current; *v* is welding speed; and, *η* is arc efficiency.

### 3.2. Residual Stresses and SCC Procedure

After repair welding, the metallographic test block is prepared from the repaired specimen. The microstructure of the weld, fusion line, and heat affected zone of metallographic specimen was observed by optical microscope. After observing the metallographic structure, the microhardness of the weld, fusion line, and HAZ of each sample was measured by Vickers hardness tester.

The residual stresses were measured by the X-ray diffraction technique. The X-ray diffraction measurement is based on Bragg’s law [50]:

(7) 2dsinθ=nλ

where *λ* is the wavelength, *d* is the interatomic lattice spacing, *n* is an integer, and *θ* is the diffraction angle.

The test method is 2*θ* – sin^2^*θ*, and the residual stress is calculated by [50]:

(8) σ=K⋅M

(9) $$K=-\frac{E}{2(1+v)}\cot\theta_{0}\frac{\pi }{180}$$

(10) $$M=\frac{\partial (2\theta )}{\partial (\sin^{2}\theta )}$$

where *K* is the stress constant, *θ*_0_ is the diffraction angle under a stress-free state, and *θ* is the angle between the normal of the crystal surface and the material surface. A linear relationship exists between 2*θ* and sin^2^*θ*, and *M* is the slope between diffraction angle 2*θ* and sin^2^*θ*. *M* is calculated if more than three points (2*θ*, sin^2^*θ*) are determined.

**Figure 4:**
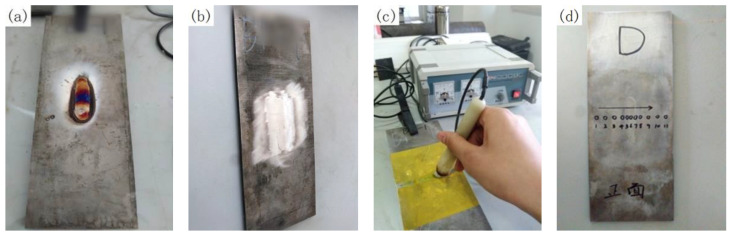
Specimens pre-treatment before X-ray diffraction (XRD): (**a**) before polished; (**b**) after polished; (**c**) electrochemical polishing; and (**d**) labeling measure point.

Before the measurement of residual stresses, the weld should be ground and leveled with a grinding wheel to remove the reinforcement height and oxide layer, as shown in Figure 4a,b. The electrolytic polishing machine is then used for electrolytic polishing along the path of measuring point to remove the grinding influence layer of grinding wheel, as shown in Figure 4c, and the polishing depth is 200 μm. Finally, the position of the measuring point should be marked on the specimen in advance. The X-ray diffraction (XRD) measurement was done by the X-350A type X-Ray stress gauge (Stress Technologies company, Handan, China). The voltage and current of the device in operation are 20 kV and 5 mA, respectively. Eleven points along P1 with an interval of 5 mm in weld and 10 mm in other area were measured, as shown in Figure 4d. Here, the path P1 is located in the center line of the upper surface perpendicular to the weld. P2 is located in the center line of the weld upper surface parallel to the weld. P3 is located in the upper surface of HAZ parallel to the weld.

**Figure 5:**
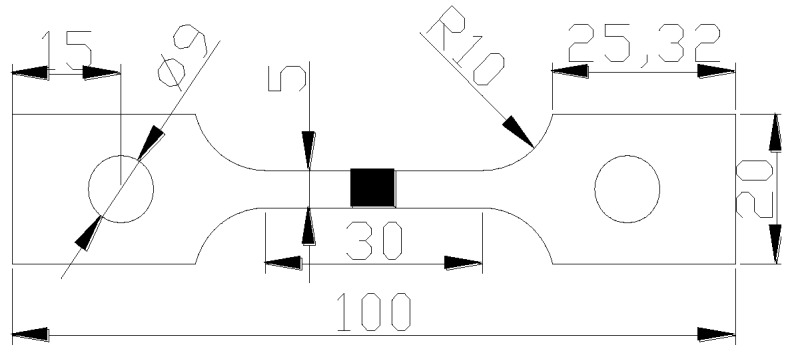
The geometry of slow strain rate tests (SSRT) specimen.

After the residual stress measurement, the test sample of slow strain rate tests (SSRT) is processed by wire cut electrical discharge machining (WEDM). Figure 5 shows the processing geometry. The tensile direction of SSRT specimen is perpendicular to the length of the repair weld, and the weld is located at the center of the SSRT specimen. Two SSRT samples were machined from the same repair welding sample, one for the tensile test in air (A1-1, A4-1) and the other (A1-2, A4-2) for the tensile test in corrosive medium.

**Figure 6:**
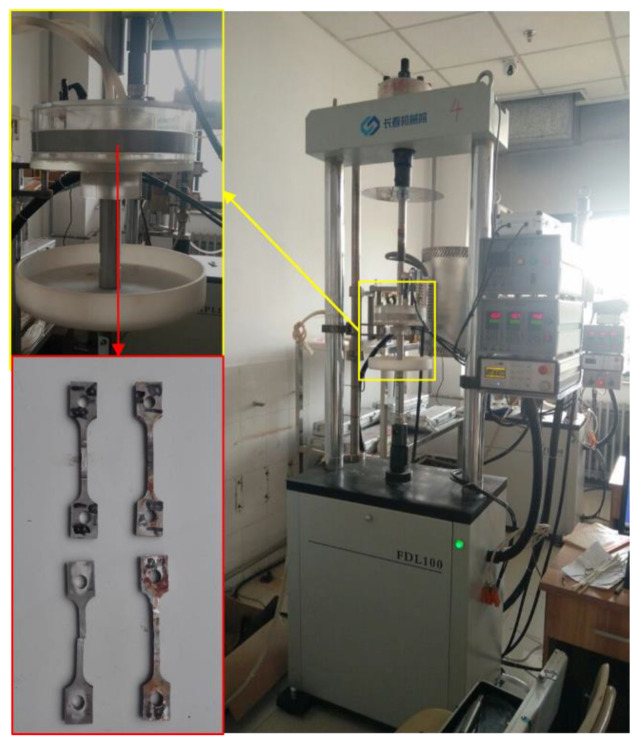
Slow strain tensile test machine.

Figure 6 shows the SSRT machine. The corrosion solution is the 3.5% NaCl solution (wt. %). The strain rate is 10^−6^ s^−1^. Before the SSRT test, the SSRT sample was ground with sandpaper to remove the residual knife mark in the process, and then rinsed with distilled water. A 300 N pre-load is applied in advance to eliminate the clearance.

The SCC sensitivity index *I_δ_* is calculated by:

(11) $$I_{\delta }=(1-\frac{\delta_{cor}}{\delta_{air}})\times 100\%$$

where *δ_cor_* and *δ_air_* is the specimen elongation in inert medium and air, respectively. The higher the sensitivity index of SCC, the greater the SCC sensitivity of the specimen. After fracture test, the fracture surface of the specimen was observed by scanning electron microscope (SEM).

## 4. Validation

**Figure 7:**
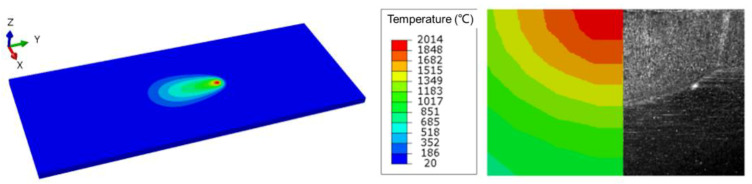
Comparison between the simulated and experimented weld pool shape.

Figure 7 compares the temperature field of the weld section by simulation and experiment. It is found that the simulated weld pool profile is roughly consistent with the actual situation. The temperature in weld center reaches 2014 °C and the temperature of the whole fusion zone has exceeded the melting point of stainless steel. The results show that the simulation method of temperature field in repair welding is feasible.

**Figure 8:**
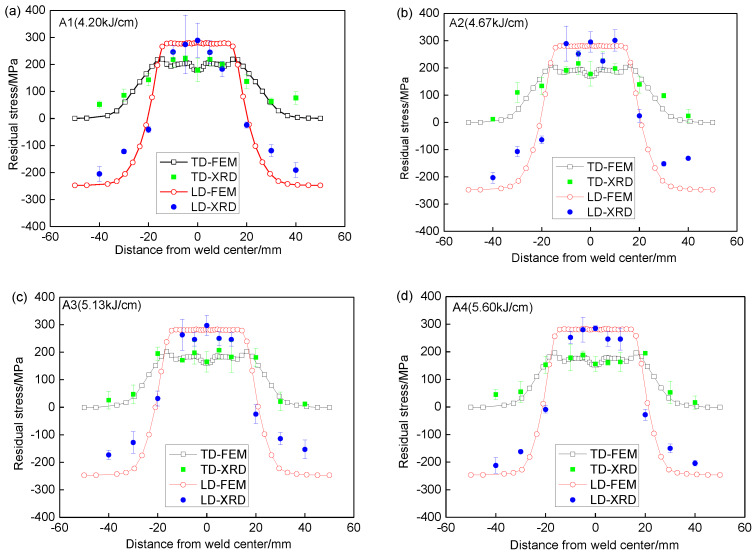
Comparison of residual stresses along P1 by simulation and measurement. (**a**) A1-4.2 kJ/cm, (**b**) A2-4.67 kJ/cm, (**c**) A3-5.13 kJ/cm, (**d**) A4-5.60 kJ/cm.

Figure 8 shows the comparison of residual stresses along P1 by simulation and measurement. It can be seen that the distribution trend of residual stresses along P1 has good agreement with that by experiment. The residual stresses in weld and HAZ are greatly larger than those in other areas. For every heat input, the longitudinal residual stresses (TD) are larger than transverse stresses (TD). The maximum error between simulation and measurement in weld and HAZ is below 9%, which proves that the simulation method on residual stress is available.

## 5. Results and Discussion

### 5.1. OM Analysis

**Figure 9:**
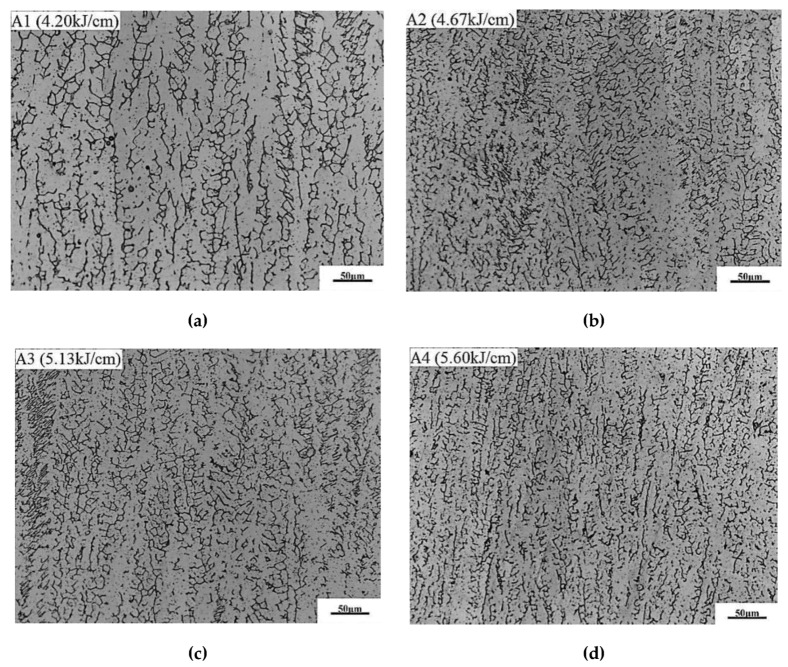
Effect of heat input on the microstructure in weld center. (**a**) A1—4.2 kJ/cm, (**b**) A2—4.67 kJ/cm, (**c**) A3-5.13 kJ/cm, (**d**) A4—5.60 kJ/cm.

Figure 9 shows the effect of heat input on the microstructure in weld. The microstructure in the weld is a composite of austenitic matrix and δ-ferrite. With the increase of heat input, the distribution of δ-ferrite is more uniform and the average grain width decreases. The ferrite content by quantitative metallography analysis for specimen A1, A2, A3, and A4 are 17.77%, 17.34%, 17.01%, and 16.27%, respectively. With the increase of heat input, the δ-ferrite in weld decreases slightly. The δ-ferrite in austenitic stainless steel weld is formed in a crystallization process and retained at room temperature. When the heat input increases, the retention time of weld metal at high temperature becomes longer, thus leading to the content of δ-ferrite in the weld decreasing. A certain content of δ-ferrite can prevent hot cracks and improve the mechanical properties of weld [51]. At the same time, the δ-ferrite can improve the resistance of intergranular corrosion and stress corrosion of weld. An excessive content of δ-ferrite will lead to embrittlement of austenite stainless steel.

**Figure 10:**
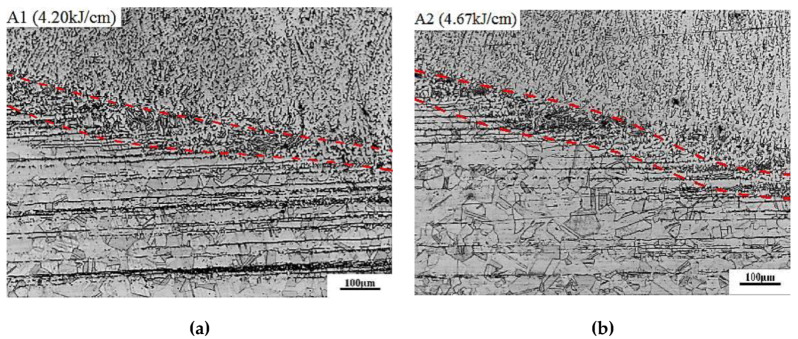

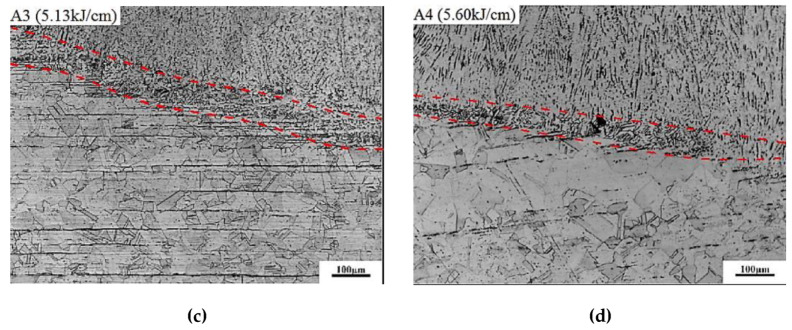
Effect of heat input on the microstructure in the fusion line and adjacent region. (**a**) A1—4.2 kJ/cm, (**b**) A2—4.67 kJ/cm, (**c**) A3—5.13 kJ/cm, (**d**) A4—5.60 kJ/cm.

Figure 10 shows the effect of heat input on the microstructure in the fusion line and the adjacent region. With the increases of heat input, the width of fusion line merely has no changes, while the austenite grain size in HAZ is slightly increased, and the strip-shaped δ-ferrite content in the rolling direction is obviously reduced. This is because the strip-shaped δ-ferrite in HAZ is the residual ferrite phase at high temperature in the forging process of steel, which can be properly eliminated by solid solution treatment. When the heat input increases, the retention time of the metal near the fusion line becomes longer at a high temperature, which is equivalent to the prolongation of the holding time of solid solution treatment, thus leading to the decrease of strip-shaped δ-ferrite content in HAZ [52].

**Figure 11:**
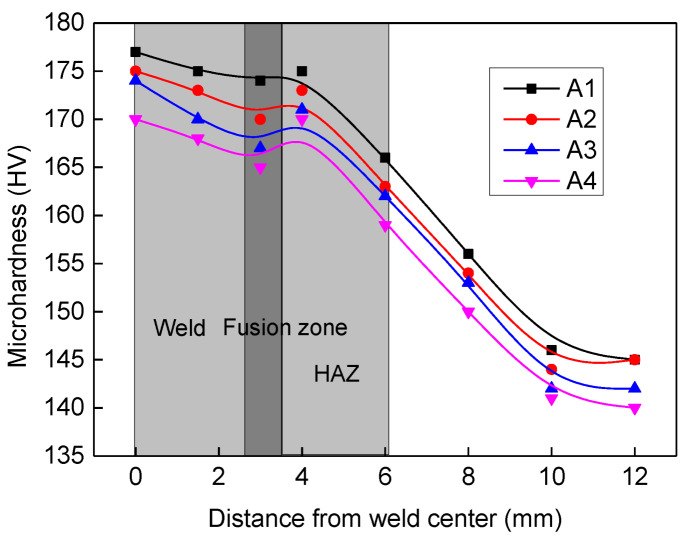
Effect of heat input on the micro-hardness for different zones.

Figure 11 shows the effect of heat input on the micro-hardness of weld, fusion line, and HAZ. The micro-hardness in all three regions is about between 160–180 HV, which is larger than that of base metal (135–160 HV). The microhardness of the fusion zone is smaller than that of weld and HAZ, because of non-uniform distribution of materials and chemical compositions. With the increase of heat input, the micro-hardness of weld and HAZ decreases slightly due to the slight decrease of δ ferrite content and the slight increase of austenite grain size.

### 5.2. Residual Stress Analysis

**Figure 12:**
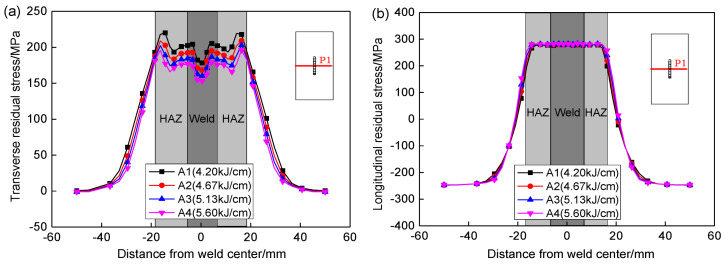
Effect of heat input on the residual stresses along P1. (**a**) Transverse stresses, (**b**) Longitudinal stresses.

Figure 12 shows the effect of heat input on the transverse and longitudinal residual stress distribution along P1 by finite element simulation. It shows that the transverse residual stresses in the weld and HAZ is gradually decreased with the increases of heat input. When the heat input is increased from 4.20 to 5.60 kJ/cm, the maximum transverse residual stress along P1 is decreased from 220 to 196 MPa, and the maximum value is reduced by about 11%. The heat input hardly affects the longitudinal residual stress, and the longitudinal residual stress in the weld and HAZ is basically maintained at approximately 280 MPa.

**Figure 13:**
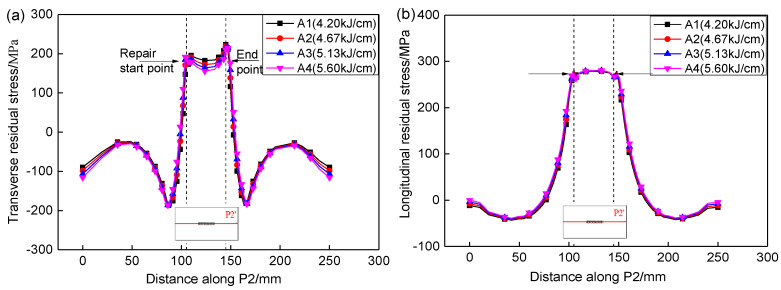
Effect of heat input on the residual stresses along P2. (**a**) Transverse stresses, (**b**) Longitudinal stresses.

Figure 13 shows the effect of heat input on the residual stresses along P2. It can be seen that the transverse residual stress at the starting point of the repair welding is less than that at ending point, while the longitudinal residual stress is basically the same at the starting and ending point. This is because the starting point of the repair welding is first heated, which is equivalent to the subsequent weld having a heat treatment effect on starting point. At the same time, the repair welding specimen is not horizontally constrained, so that the transverse residual stress at the starting point of the repair welding is partly released, and finally leads to the transverse residual stress at the beginning of repair welding is less than that at end point of repair welding. The longitudinal residual stress at the starting point of the repair welding can not be released during repair heating, due to the large constraint in the longitudinal direction, thus leading to the longitudinal residual stress at start point is the same as that at ending point. There is a great transverse stress gradient at the start and end point of repair weld. The transverse and longitudinal residual stresses were increased from compressive stress to tensile stress along the entire length P2. With the increases of heat input, the transverse residual stresses in weld were gradually decreased, while the longitudinal stresses merely have no changes. That is because the peak residual stresses are determined by the yield strength instead of the heat input. The longitudinal stresses in the weld and HAZ have reached the yield strength, thus the heat input affects them little.

**Figure 14:**
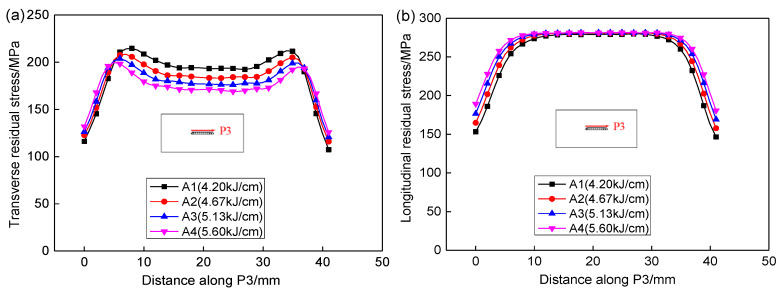
Effect of heat input on the residual stresses along P3. (**a**) Transverse stresses, (**b**) Longitudinal stresses.

Figure 14 shows the effect of heat input on the residual stresses in HAZ along P3. Similar to the residual stress in weld, the transverse residual stress was decreased as heat input increases and the longitudinal stress in HAZ has no changes. When the heat input was increased by 33%, the average transverse stress along P3 in weld and HAZ decreased by 30% and 35%, respectively.

### 5.3. SCC Sensitivity Analysis

**Figure 15:**
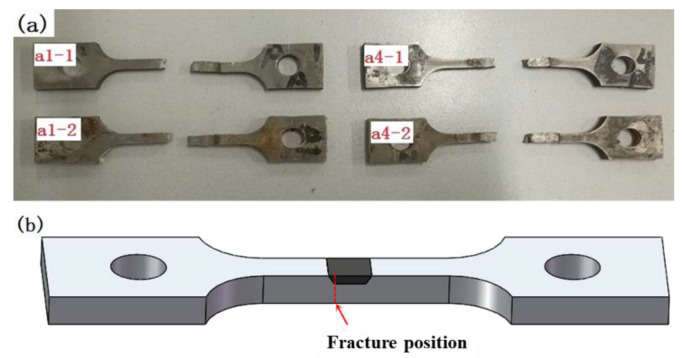
The fractured SSRT specimens (**a**) and fracture position (**b**).

Figure 15 shows the fractured SSRT specimens and fracture position. All of the specimens were fractured on the side of the original defect, as shown in Figure 15b, which indicated indicating that the original defect edge of the repair welding is the dangerous position to cause the equipment to crack again. The inhomogeneous materials and residual stress might induce this.

**Figure 16:**
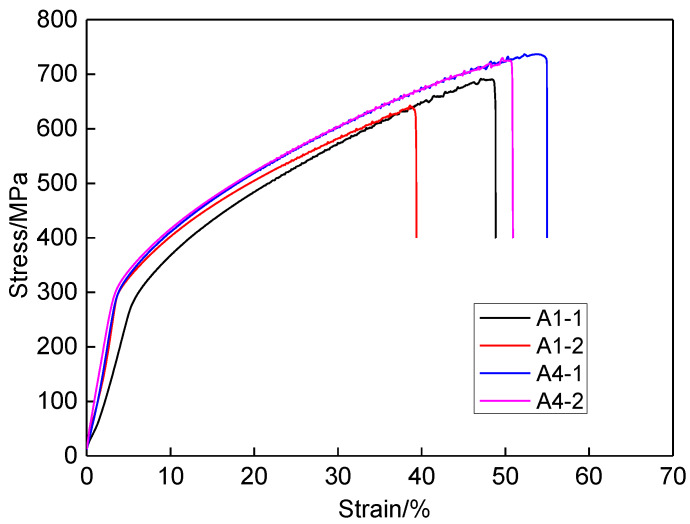
The stress-strain curve of SSRT test.

Figure 16 show the stress-strain curve of SSRT test for different specimens. Undoubtedly, the tensile strength and elongation in corrosive solution is smaller than that in air. Whether in air or corrosive solution, the tensile strength and elongation for larger heat input is larger than those for smaller heat input. The higher heat input can enhance the strength and mechanical properties of repaired joint. Table 3 lists the tensile strength, fracture time, and elongation for different SSRT specimens. According to Equation (6), the SCC sensitivity index can be obtained, as listed in Table 4. The SCC sensitivity index is smaller than 25%, indicating the repaired joint has no obvious SCC tendency. The SCC sensitivity index for heat input 5.60 kJ/cm (7.53) is greatly smaller than that for heat input 4.20 kJ/cm (19.37). When the heat input was increased by 33%, the SCC sensitivity index was decreased by more than 60%. Therefore, increasing the heat input can enhance the resistance ability to stress corrosion of repaired joint.

**Figure 17:**
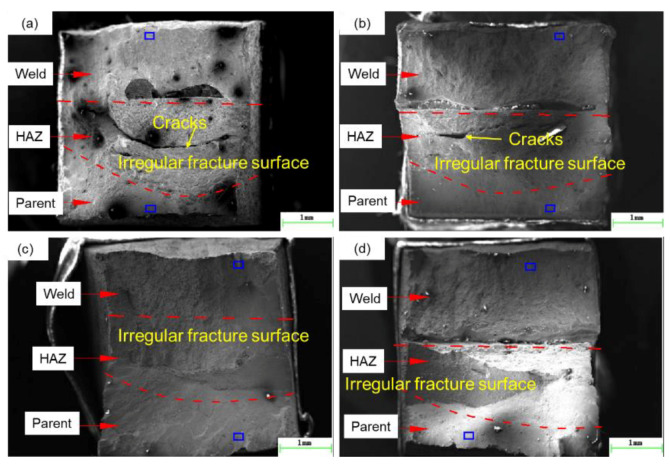
The macro fracture surface morphology for the specimen (**a**) A1-1, (**b**) A1-2, (**c**) A4-1, and (**d**) A4-2.

Figure 17 shows the macro fracture surface morphology of SSRT specimens. The fracture morphology is different for different specimens. The fracture surface of weld zone and base metal is smooth, while the fracture surface of HAZ is irregular, which is composed of several grain inclined planes in different directions. There are macroscopic cracks in the HAZ for specimen A1-1 and A1-2, and no macroscopic cracks are found in the HAZ for specimen A4-1 and A4-2. The macroscopic cracks are easily generated in HAZ for the smaller heat input, leading to the smaller tensile strength and elongation.

**Figure 18:**
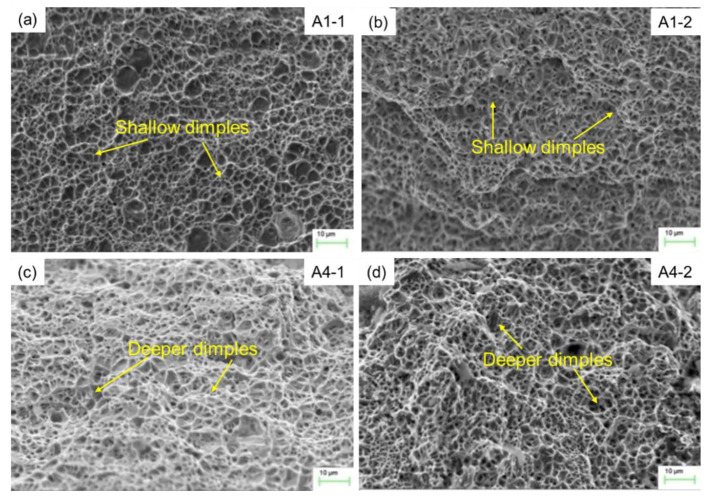
The micro fracture surface morphology in weld for the specimen (**a**) A1-1, (**b**) A1-2, (**c**) A4-1, and (**d**) A4-2.

The micro fracture surface morphology in weld by SEM is shown in Figure 18 in order to reveal the effect of heat input on fracture mechanism in weld. It is found that the microfracture surfaces in weld of the four specimens show dimple fracture characteristics. For the specimen A1-1 and A1-2, the dimples are shallow and smaller than that for the specimen A4-1 and A4-2. The higher the heat input, the deeper and bigger fracture dimples, which results in better mechanical properties.

Based on the above studies, we can conclude that a higher heat input is beneficial for reducing residual stress and enhancing microstructure, mechanical properties of repair joint. Heat input is an essential factor in quality control in repair welding. It influences the cooling rate after welding, which affects the mechanical properties and microstructure of the weld and HAZ and, consequently, affects the residual stress distribution. With the heat input increases, the maximum temperature at weld pool is increased. On a micro scale, the δ-ferrite in weld and the average grain width decreases slightly with the increases of heat input, because the retention time of weld pool at a high temperature becomes longer. On the macro scale, the larger heat input leads to the increases of plastic zone, then resulting in the more residual stresses are released, which is similar to the results that were found by Jiang [15] and Unnikrishnana [33]. The tensile residual stress is main factor for resulting in SCC of welded joint at corrosive environment. The larger the residual stress, the easier to occur SCC. Thus, the SCC sensitivity index was decreased for the larger heat input, which was also verified by Mohammed [32]. To sum up, the larger heat input will be beneficial to improve the entire performance of welded joints, including strength, microstructure, corrosion resistance, etc. In reality, factories often meet production schedules by providing higher heat input to achieve higher welding speeds. Here, in this study, the heat input larger than 5 KJ/cm is recommended in the repair welding in 304 stainless steel in the case of no welding defects.

## 6. Conclusions

In this paper, the effect of repair welding heat input on microstructure, residual stresses, and SCC sensitivity were investigated by simulation and experiment. An optimized repair welding heat input was proposed. The main conclusions are drawn, as follows:

(1) The heat input influences the microstructure. With the increase of heat input, the δ-ferrite in weld and the average grain width decrease slightly, while the austenite grain size in HAZ is slightly increased. The heat input has little effect on the micro-hardness.

(2) With the increases of heat input, the transverse residual stresses in the weld and HAZ is gradually decreased, while the longitudinal stresses merely have no changes. When the heat input was increased by 33%, the average transverse stresses were decreased by more than 30%.

(3) All of the specimens under SSRT were fractured on the side of the original defect. The higher heat input can enhance the tensile strength and elongation of repaired joint. When the heat input was increased by 33%, the SCC sensitivity index was decreased by more than 60%.

(4) A different heat input has a different fracture mode in weld and HAZ. The macroscopic cracks are easily generated in HAZ for the smaller heat input, leading to the smaller tensile strength and elongation. The heat input larger than 5KJ/cm is recommended in the repair welding in 304 stainless steel.

## Figures and Tables

**Table 1:** Chemical compositions of 304 stainless steel (wt%).

C	Si	Mn	P	S	Cr	Mo	Ni	Cu	Fe
0.048	0.419	1.228	0.031	0.002	18.08	0.011	8.113	0.009	Bal.

**Table 2:** The temperature-dependent thermal physical properties of 304 stainless steel.

Temperature (°C)	20	200	400	600	800
Poisson’s ratio	0.28	0.28	0.28	0.28	0.28
Density (kg/m^3^)	8010	7931	7840	7755	7667
Specific heat (J/kg °C)	500	544.3	582	634	686
Young’s Modulus (GPa)	199	180	166	150	125
Yield strength (MPa)	206	153	108	82	69
Conductivity (W/m °C)	15.26	17.6	20.2	22.8	25.4
Thermal expansion coefficient (1/°C ×10^−6^)	16.0	17.2	18.2	18.6	19.5

**Table 4:** The mechanical parameters and SCC sensitivity index of SSRT test.

Specimen No.	Tensile Strength *R*_m_/MPa	Fracture Time *t*/h	Elongation *δ*/%	SCC Sensitivity Index *I_δ_*/%
A1-1	691.64	196.62	48.83	19.37
A1-2	642.72	158.32	39.37	
A4-1	737.04	218.22	54.96	7.53
A4-2	729.76	201.78	50.82	

**Table 3:** Welding parameters of repaired specimens with different heat inputs.

Specimen No.	Repair Length	Voltage	Current	Welding Speed	Heat Input
	*L*/mm	*U*/V	*I*/A	*v*/(mm/s)	*q*/(kJ/cm)
A1	40	20	90	3	4.20
A2	40	20	100	3	4.67
A3	40	20	110	3	5.13
A4	40	20	120	3	5.60
